# Synthesis and Characterization of a New Alginate/Carrageenan Crosslinked Biopolymer and Study of the Antibacterial, Antioxidant, and Anticancer Performance of Its Mn(II), Fe(III), Ni(II), and Cu(II) Polymeric Complexes

**DOI:** 10.3390/polym15112511

**Published:** 2023-05-30

**Authors:** Yassine EL-Ghoul, Maged S. Al-Fakeh, Nora S. Al-Subaie

**Affiliations:** 1 Department of Chemistry, College of Science, Qassim University, Buraidah 51452, Saudi Arabia; m.alfakeh@qu.edu.sa (M.S.A.-F.); 411200095@qu.edu.sa (N.S.A.-S.); 2 Textile Engineering Laboratory, University of Monastir, Monastir 5019, Tunisia; 3 Taiz University, Taiz 3086, Yemen

**Keywords:** alginate, carrageenan, biopolymer complex, antimicrobial, antioxidant, anticancer

## Abstract

Natural polysaccharides are essential to a wide range of fields, including medicine, food, and cosmetics, for their various physiochemical and biological properties. However, they still have adverse effects limiting their further applications. Consequently, possible structural modifications should be carried out on the polysaccharides for their valorization. Recently, polysaccharides complexed with metal ions have been reported to enhance their bioactivities. In this paper, we synthesized a new crosslinked biopolymer based on sodium alginate (AG) and carrageenan (CAR) polysaccharides. The biopolymer was then exploited to form complexes with different metal salts including MnCl_2_·4H_2_O, FeCl_3_·6H_2_O, NiCl_2_·6H_2_O, and CuCl_2_·2H_2_O. The four polymeric complexes were characterized by Fourier-transform infrared spectroscopy (FT-IR), elemental analysis, ultraviolet–visible spectroscopy (UV–Vis), magnetic susceptibility, molar conductivity methods, and thermogravimetric analysis. The X-ray crystal structure of the Mn(II) complex is tetrahedral and belongs to the monoclinic crystal system with the space group P121/n1. The Fe(III) complex is octahedral and crystal data fit with the cubic crystal system with the space group Pm-3m. The Ni(II) complex is tetrahedral and crystal data correspond to the cubic crystal arrangement with the space group Pm-3m. The data estimated for the Cu(II) polymeric complex revealed that it is tetrahedral and belongs to the cubic system with the space group Fm-3m. The antibacterial study showed significant activity of all the complexes against both Gram-positive bacteria (*Staphylococcus aureus* and *Micrococcus luteus*) and Gram-negative (*Escherichia coli* and *Salmonella typhimurium*) pathogenic strains. Similarly, the various complexes revealed an antifungal activity against *Candida albicans*. The Cu(II) polymeric complex recorded a higher antimicrobial activity with an inhibitory zone reaching 4.5 cm against *Staphylococcus aureus* bacteria and the best antifungal effect of 4 cm. Furthermore, higher antioxidant values of the four complexes were obtained with DPPH scavenging activity varying from 73 to 94%. The two more biologically effective complexes were then selected for the viability cell assessments and in vitro anticancer assays. The polymeric complexes revealed excellent cytocompatibility with normal human breast epithelial cells (MCF10A) and a high anticancer potential with human breast cancer cells (MCF-7) which increase significantly in a dose-dependent manner.

## 1. Introduction

In recent years, the bioresource sector has experienced rapid progress, particularly with the increased exploitation of algae as a biomass feedstock. Algae have the potential to generate a variety of naturally occurring compounds including carbohydrates, polysaccharides, lipids, proteins, oils, and other bioproducts. The low-cost and sustainable nature provides algae with an efficient tool for producing a multitude of bioproducts [1,2,3]. Algae are an alternative natural source for the production of a wide variety of renewable biopolymers. These seaweed biopolymers have proven excellent properties and a broad spectrum of applications. They are used directly or modified after chemical functionalization or mixing and as material reinforcement in many medical, food, cosmetic, and environmental fields [4,5,6,7,8,9]. Different natural polysaccharides are extracted from algae biomass, such as alginate, carrageenan, laminarin, fucoidan, agar, etc. They exhibit various biological properties. Indeed, they are biocompatible, less toxic, and have revealed significant antimicrobial, antioxidant, antitumor, and immunomodulatory activities [10,11,12,13,14]. Algae-derived bio-organic matter provides a wide range of various polyanions, including alginate biopolymers. Alginate (AG) is a natural polysaccharide composed of β-D-mannuronic acid and α-L-guluronic acid, obtained efficiently by alkaline extraction from the cell wall of brown algae [15]. It is characterized by high ion selectivity and a strong tendency to form heterogeneous and homogeneous polymer associations. Owing to its high-stability thickening and gelling characteristics and excellent properties such as biocompatibility, biodegradability, immunogenicity, antibacterial activity, hydrophilicity, and non-toxicity, alginate is considered an important industrial polysaccharide. Therefore, AG is applied in various fields, including food, packaging, composites, cosmetics, textile printing, medicine, biomaterials, and pharmaceuticals [16,17,18,19,20]. Carrageenan is a linear sulfated polysaccharide and a major constituent of red edible seaweeds. The additional sulfate groups in the repeating galactose units are behind the chemical reactivity of carrageenan. Their number and position allow carrageenan to be classified into three major commercially relevant families, namely kappa (k), iota (i), and lambda (l). It is extensively exploited in the food industry, due to its thickening, gelling, and stabilizing characteristics. Recently carrageenan biopolymer has shown excellent capacities in different applied fields such as biomaterials, drug delivery, medicine, packaging, and pollutant adsorption [21,22,23,24,25]. The macromolecular chains of these natural polysaccharides are considered effective in chelating polyvalent metal ions. However, it should be noted that, despite the various confirmed benefits, alginate and carrageenan still present some deficiencies and adverse effects on biological systems, and this could be overcome by providing certain functionalizations with natural and synthetic polymers or designing some co-ordination polymers and complex metal frameworks [26,27,28]. The synthesis of co-ordination polymers, mainly biomineralizing systems, has recently experienced rapid development in biochemistry and materials science. Biomineralization systems exhibit some desirable and relevant characteristics in their ability to elaborate nanoscale clusters and orderly assemble organized inorganic phases with different biopolymeric polysaccharides. A great deal of attention has been given to these hybrid organo-mineral systems due to the novel additional properties that go beyond the usual characteristics of the minerals themselves. The complexation of different organic materials with metal ions provides them with new biological properties which are emerging as an important path toward the development of new drugs. There is ample evidence that the incorporation of metal ions into bioactive substances amplifies their effect by altering the electronic and geometrical composition [29,30,31,32]. Polysaccharides can be associated with metal ions by different mechanisms, including ion exchange, sorption, and chelation. Previous literature has shown the formation of amorphous metal assemblies in certain assemblies of proteins, alginate, sulfonated dextrans, carrageenan, chitosan, and other natural polymers [33,34,35,36,37,38]. In the present research work, we sought to take advantage of the benefits of polyanionic alginate and carrageenan biopolymers by crosslinking them using citric acid to obtain a copolymer with more binding sites and improved biological applied properties. After optimizing the condition of their crosslinking, the resulting polymer will be characterized using different techniques. Then, different transition and non-transition metal mixed ligand co-ordination polymers will be synthesized using Mn(II), Fe(III), Ni(II), and Cu(II) metals. Afterward, a study of the physical, chemical, thermal, morphological, and biological activity of the made co-ordination complexes will be performed. Finally, cell viability assays using normal human breast epithelial cells (MCF10A) and human breast cancer cells (MCF-7) will be performed to assess the cytocompatibility and the anticancer potential of the prepared polymeric complexes.

## 2. Experimental

### 2.1. Materials

Sodium alginate (AG) with a degree of deacetylation of 70%, medium viscosity, and an average molecular weight of about 30,000 g/mol was purchased from Sigma-Aldrich (St. Louis, MO, USA). Citric acid used as cross-linking agent (CTR, 226.2 g/mol) and iota-carrageenan (CAR, a fine white powder) were supplied by Sigma-Aldrich. All chemicals were of analytical grade and used as received without further purification.

### 2.2. Characterization of the Polymer Complexes

An elemental analysis, CHN, of the different co-ordination polymer complexes, was carried out via an Analytischer Functions test elemental analyzer (VELP Scientifica Srl, Usmate (MB), Italy).

FTIR spectra using the attenuated total reflection (ATR) mode were performed using an infrared spectrophotometer (Agilent technologies/Gladi-ATR, Santa Clara, CA, USA). IR Spectra were recorded in a wavenumber range varying from 4000 to 400 cm^−1^. The resolution employed for the different measurements was fixed to 4 cm^−1^. A magnetic susceptibility balance (MSB-Auto) from Sherwood Scientific (Cambridge, UK) was used to measure the magnetic moments of the different prepared polymer complexes. A UV–Vis spectrophotometer (Shimadzu, UV-2101PC, Kyoto, Japan) was used to measure the UV–vis absorption curves of the different polymer complexes. The wavelength range varied from 200 to 700 nm for the different measurements. A Jenway benchtop conductivity meter (model 4310, London, UK) was used to determine the conductance for the synthesized co-ordination polymers. Thermogravimetric analysis was performed via Shimadzu thermal analyzer (DTG 60-H, Kyoto, Japan) under a heating rate of 10 °C/min at a temperature range of 20–600 °C. The X-ray diffraction patterns were determined using an X-ray Diffractometer (PW 1720 Philips, Eindhoven, The Netherlands). The polymer complex samples were analyzed at a scan range of 10–90°, and the step width was 0.01°. Scherrer’s Equation (1) was applied to estimate the average crystal particle size of the co-ordination polymers:

(1) $$D=\frac{K\lambda }{\beta cos\theta }$$

where *K* is the form factor, *λ* is the X-ray wavelength (typically 1.54 Å), *β* is the line widening at half the maximum intensity in radians, *θ* is the Bragg angle, and *D* is the average size of the ordered (crystalline) domains, which can be less than or equal to the grain size. In order to compare Bragg intensities with those calculated from a possible structural model, the approach uses a least squares procedure. The global parameters, including background and scale factors, were refined in the initial stage of the process. The structural parameters were then sequentially refined in the following stage, including the lattice parameters, profile shape and width parameters, preferred orientation, asymmetry, isothermal parameters, atomic coordinates, and site occupancies. By computing indicators such as the goodness of fit “χ^2^” and the R factors (R_wp_ = weighted profile R-factor, R_B_ = Bragg factor, and R_exp_ = expected R factor), the fitting quality of the experimental data is evaluated. 

The surface morphological behavior of the different elaborated complex systems was accomplished using scanning electron microscopy (JEOL JSM-5400 LV, JEOL Ltd., Akishima, Japan). Different micrographs were recorded under an acceleration voltage of 5 kV. The magnification scale ranged from 100 to 3000×. A thin layer coating of Au was applied for the different samples before the SEM analysis with the aim of improving surface conductivity and thus obtaining clearer images.

### 2.3. Preparation of the Crosslinked Polymeric Ligand (Poly-AG/CTR/CAR)

Firstly, in a round-bottom flask, we dissolve 2 g of sodium alginate in 100 mL of distilled water under vigorous stirring for 1 h at 80 °C. Then, we added dropwise a solution of carrageenan previously prepared by dissolving 2 g of carrageenan in 100 mL of distilled water and heating at 70 °C under continuous stirring. The mixture was kept stirring for 1 h. After that, we added 2 g of citric acid used as a crosslinking agent. The preparation was stirred at 120 °C for 2 h, under N_2_ pressure. The reaction solution was then kept under stirring at 90 °C overnight. Then, the resulting suspension was cooled to room temperature before being concentrated under reduced pressure. Finally, the obtained product was dried at 60 °C under a high vacuum to yield a white stable powder (90%). 

### 2.4. Preparation of Polymer/Metal Complexes

#### 2.4.1. Preparation of the Poly-AG/CTR/CAR/Mn(II) Complex

First, we prepare 0.2 g of MnCl_2_·4H_2_O dissolved in 15 mL of distilled water (solution 1). At the same time, a second solution (Solution 2) is prepared, containing 0.8 g of the ligand polymer dissolved in a mixture of ethanol/water (10/10 mL). Solution 2 is subjected to heating at 70 °C for 15 min. After cooling to ambient temperature, we add Solution 1 of the metal under continuous stirring for 3 h. Finally, we collect in a Petri dish by filtration the formed precipitate which will be dried in an oven for two days at 50 °C. The final covered product is a fine powder with a light brown color.

#### 2.4.2. Preparation of the Poly-AG/CTR/CAR/Fe(III) Complex

In the same way as before, the first solution (Solution 1) is prepared by dissolving 0.6 g of FeCl_3_·6H_2_O in 15 mL of distilled water. We leave Solution 1 under stirring and we prepare the solution of the polymeric ligand (Solution 2) by dissolving 0.8 g in an ethanol/water mixture (10/10 mL). The solution is heated at 70 °C. for 15 min with continuous stirring. After cooling to ambient temperature, Solution 1 of the metal is added with continuous stirring for 3 h. A precipitate is formed at the bottom of the round-bottom flask which will be collected and dried in an oven for 24 h at 50 °C. The final product is a fine powder of light orange color.

#### 2.4.3. Preparation of the Poly-AG/CTR/CAR/Ni(II) Complex

In a first round-bottom flask, we dissolve 0.35 g of NiCl_2_·6H_2_O in 15 mL of distilled water (Solution 1) and stir at room temperature. During this time, the solution of the polymeric ligand is prepared (Solution 2) by dissolving 0.8 g in an ethanol/water mixture (10/10 mL). Solution 2 is heated at 70 °C. for 15 min with continuous stirring. After cooling, we add Solution 1 containing the metal with continuous stirring for 3 h. A precipitate is formed at the bottom of the round-bottom flask which will be collected and dried in an oven for 48 h at 50 °C. The final product obtained is a fine powder of light green color.

#### 2.4.4. Preparation of the Poly-AG/CTR/CAR/Cu (II) Complex

We start by dissolving 0.30 g of CuCl_2_·2H_2_O in 15 mL of distilled water (Solution 1) and let it stir at room temperature. Simultaneously, a solution containing 0.8 g of the polymeric ligand in an ethanol/water mixture (10/10 mL) is prepared (Solution 2). After heating at 70 °C for 15 min with continuous stirring, Solution 2 is cooled and we add Solution 1 containing the metal. The mixture is carried out with continuous stirring for 3 h. Finally, the precipitate formed at the bottom of the round-bottom flask is collected by filtration and dried in an oven for 48 h at 50 °C. The final product obtained is a light blue fine powder.

### 2.5. Biological Analysis

#### 2.5.1. Antimicrobial Activity

A Mueller–Hinton agar disk diffusion method was used to determine the antimicrobial activity of the different polymeric complexes [39]. Bacteriological analyses were performed using two Gram-positive bacteria, Staphylococcus aureus (Sa, ATCC 25923) and Micrococcus luteus (Ml, NCIMB 8166), and two Gram-negative bacterial strains, Escherichia coli (Ec), and Salmonella typhimurium (St). The antifungal test was accomplished against a pathogenic reference strain of the yeast Candida albicans ATCC 90028. Samples were prepared by dissolving 50 mg of each polymeric complex in 1 mL of a DMSO solution (5%). The bacteria strains were cultivated in nutrient agar, Mueller–Hinton (MH) broth (Oxoid), at 37 °C for 24 h. 

The prepared suspensions were then adjusted to 0.5 McFarland standard turbidity. Then, 100 μL of each bacterial suspension was spread on an MH agar dish and incubated at 37 °C for 30 min. Afterward, sterile filter-paper discs (6 mm in diameter) were impregnated with 20 μL of the different samples and placed on agar plates and held for 1 h at 4 °C. After incubation (24 h at 37 °C), where the tested disc-shaped samples could diffuse into the MH agar, the diameter of the inhibition area (clear halo) that appeared around the disc samples was measured. Analyses were carried out in duplicate.

#### 2.5.2. Antioxidant Activity

The antioxidant performance of the different polymeric complexes was evaluated via the DPPH free-radical scavenging assay according to the method reported by Mahdhi et al. [40]. Concisely, 1 mL of each sample (5 mg/mL) was added to 3 mL of a prepared methanolic solution of DPPH (2,2-diphenyl-1-picrylhydrazyl) (300 µM). The reaction mixture was then vortexed and incubated for 30 min at room temperature. The absorbance of the solution was measured at 517 nm. Ascorbic acid was used as the standard positive control. The DPPH inhibitory effect was calculated according to the following Equation (2).

(2) $$DPPH\;Scavenging\;effect\left(\%\right)=[1-(Abs\;sample/Abs\;control)]\times 100$$

DPPH antioxidant assays were carried out in triplicate and mean values were recorded.

#### 2.5.3. Cell Viability and Anticancer Assays

For the cell culture, human normal breast epithelial cells (MCF-10A), and human breast cancer cells (MCF-7) were procured from American Type Culture Collection (Manassas, WV, USA). The cells were grown in Dulbecco Modified Eagle’s Medium with the addition of a prepared solution of fetal bovine serum (10%) and a mixture of penicillin (100 IU/mL) and streptomycin (100 μg/mL) as antibiotics. The medium was then incubated in a 5% CO_2_ atmosphere, with 100% relative humidity at 37 °C. The anticancer activity and the cell viability assays were performed via the MTT tetrazolium standardized test with some modifications [41]. This test is based on the ability of live cells to proliferate and thus metabolize and reduce the yellow MTT tetrazolium salt into purple formazan, characterized by a typical absorbance at 570 nm. The cultivated cells were seeded into 96-well plates at a density of 2 × 10^4^ cells/well. After 24 h of incubation, samples with different concentrations were added to each cell culture medium. After incubation for 72 h, we proceed with the MTT assay. Cell viability was assessed as the average percentage of relative formazan crystals formed, taking into account the control culture. Assays were carried out in triplicate for each test.

## 3. Results and Discussion

### 3.1. Preparation of the Different Polymeric Complexes

**Figure 1:**
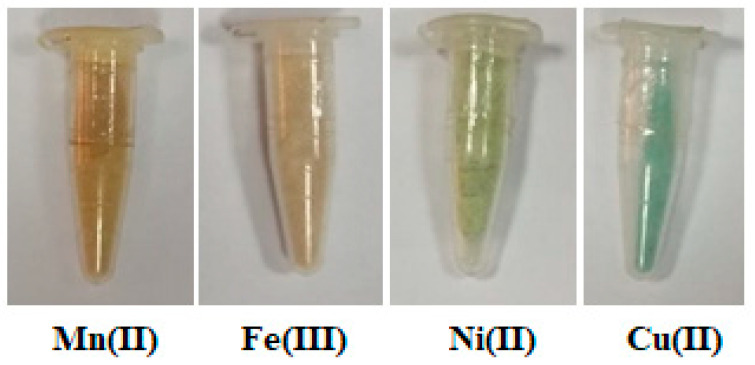
Different synthesized polymeric complexes.

The poly-AG/CTR/CAR ligand was synthesized by the reaction of the two alginate and carrageenan biopolymers in the presence of citric acid as a crosslinking agent. The reaction happened through the polyesterification reaction, on the one hand, between the CTR and hydroxyl groups of the alginate biopolymer and, on the other hand, between the carboxylic groups of the CTR and the hydroxyl groups of the carrageenan to result in the formation of the crosslinked polymer, the poly-AG/CTR/CAR. The different Mn(II), Fe(III), Ni(II), and Cu(II) coordination complexes were synthesized by reacting the poly-AG/CTR/CAR with the various metal salts. The different reactive groups of alginates, carrageenan, and CTR give the crosslinked poly-AG/CTR/CAR an effective ability to bind different metals. The four synthesized complexes were found to react in a 1:1 molar ratio (metal cation: SO_4_^−^ from CAR or COO^−^ from AG). The obtained polymer complexes revealed different specific colors as shown in Figure 1. Indeed, the color of the Mn(II) complex was light brown, the Fe(III) complex was light orange-brown, the Ni(II) was light green, and the Cu(II) was light blue. Moreover, the different complexes were stable in air and insoluble in water and in the common organic solvent except in the DMSO. For this reason, the conductivity measurement for the different complexes (10^−3^ M) was carried out in the DMSO solvent. The molar conductivity values of the metal complexes Mn(II), Fe(III), Ni(II), and Cu(II) were 2.58, 2.08, 6.78, and 2.95 Am Scm^2^ mol^−1^, respectively. The various properties of the complex biopolymers, including color, elemental analysis, decomposition point, and conductivity, are summarized in Table 1.

### 3.2. FT-IR Analysis

**Figure 2:**
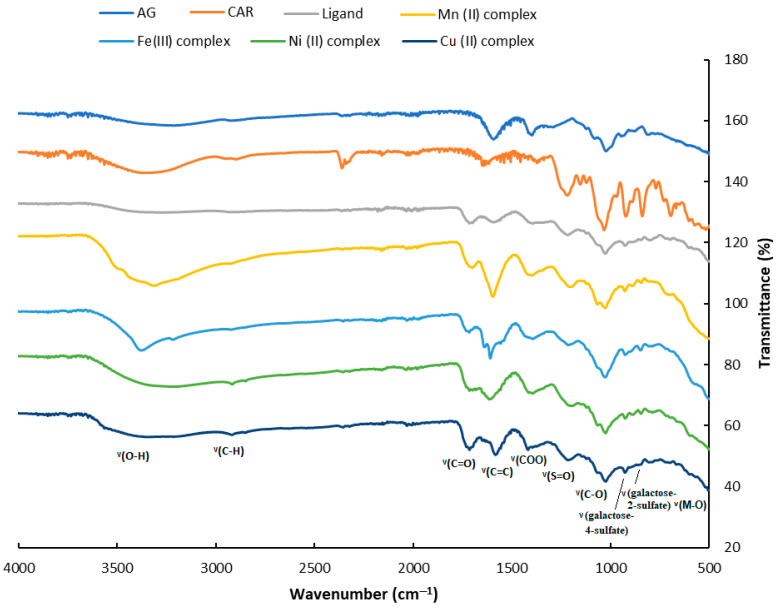
FT-IR spectra of the alginate, carrageenan, polymeric ligand (poly-AG/CTR/CAR), and the different prepared Mn(II), Fe(III), Ni(II), and Cu(II) coordination complexes.

The FT-IR spectrophotometric analysis was performed to identify the functional groups of the materials and to understand the binding mechanism. Herein, on the one hand, the IR analysis was carried out with the aim of evaluating the synthesis of the polymeric ligand via the crosslinking process, and, on the other hand, this technique would be decisive in confirming the formation of the various complexes between the polymer produced and the various metals investigated in our study. The different obtained spectra are presented in Figure 2 and the various assignments of the main peaks are summarized in Table 2. Concerning the synthesis of the polymeric ligand, from the comparison of the spectrum of the alginate or carrageenan biopolymer and that of the ligand, we notice the apparition of a new band at around 1712 cm^−1^, referring to the formation of the ester group. This ester formation was between the carboxylic groups of the CTR as a crosslinking agent and between both the hydroxyl groups of the glycosidic moiety of the alginate and carrageenan biopolymers. Therefore, we deduce that the polymeric ligand was prepared by a polyesterification reaction between the alginate and carrageenan via the intermediate of the CTR crosslinking agent. The IR spectrum of the ligand showed different characteristic bands also present in the spectra of the initial polymers. Indeed, we remark the presence of a large band at around 3431 cm^−1^ which is attributed to the hydroxyl groups stretching (O-H). A strong band near 1035 cm^−1^ showed the C-O-C stretching vibration of the glycosidic structure [42]. Absorption bands in the range of 1000–1100 cm^−1^ are assigned to the C-O-C glycosidic bonds of the pyranose—absorption bands at 783 and 560 cm^−1^. For the various spectra of the complexes compared to the polymeric ligand alone, we notice some apparent shifts, especially at 3431 (ν O-H), 1725 (ν O-H), and 1260 cm^−1^ (ν S=O). These wavenumber shifts were due to evident interactions between the metal and the related groups in the polymeric ligands. The different absorbances linked to carrageenan sulfate groups including sulfate (1214 cm^−1^), galactose-4-sulfate (920 cm^−1^), and galactose-2-sulfate (834 cm^−1^) are presented in the polymeric ligand [43]. These latter groups also appeared with different complexes revealing clear shifts towards lower wavenumber values, indicating the formation of an interaction between the different sulfate functions of the ligand and the various metals. The band appearing at 505–516 cm^−1^ with the four complexes is assigned to the (H_2_O) which confirms the presence of coordinated water (ν M-O) [44]. We can conclude that the different functional groups of the synthesized crosslinked polymer, the poly-AG/CTR/CAR, which are hydroxyl, carboxylic, ester, and sulfate groups, are able to coordinate with the various metal ions, and are thus mainly in a bidentate mode.

### 3.3. UV–Vis Spectrophotometric Analysis

Different UV–visible spectra of the complexes were recorded using DMSO as a solvent. The different absorption bands and their assignments are shown in Table 3. Apparent absorption bands appeared at 24.213 and 39.215 nm, which are attributed to the n→π* and π→π* transitions within CAR and AG biopolymers. The Mn(II) complex showed characteristic bands at 29.761 nm, 35.842 nm, and 21.551 nm, which refer to n→π*, π→π*, and 4T1g (D)→6A1g, respectively. The Fe (III) complex showed typical bands at 29.411, 32.573, and 21.598 nm, attributed to n→π*, π→π*, and 4T2g (G)→6A1g. In the case of the Ni(II) complex, three characteristic bands appeared at 23.202, 34.072, and 21.978 nm, which are assigned to n→π*, π→π*, and 3A2g (F)→3Tg. As for the Cu(II) complex, the analysis exhibited two principal bands centered at 27.548 and 35.211 nm, referring to both n→π* and π→π*. The different characteristic bands of Mn(II), Fe(III), Ni(II), and Cu(II) complexes appearing in the visible region have been assigned to d–d transitions typical of octahedral metal complexes [45,46].

### 3.4. Magnetic Moments

**Figure 3:**
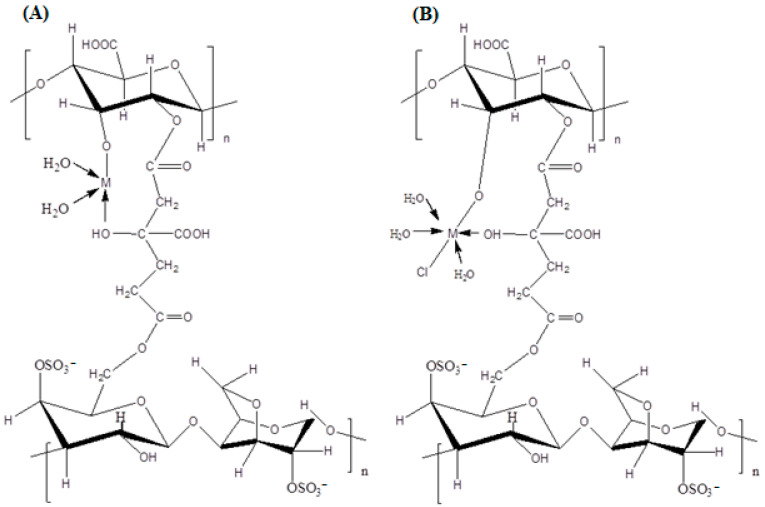
Proposed structures of the complexes (**A**) [M(AG/CTR/CAR)(H_2_O)_2_], M=Mn(II), Ni(II) and Cu(II) and the complex (**B**) [Fe(AG/CTR/CAR)(H_2_O)_3_ Cl].

Measurements of the magnetic moments of the different compounds performed at room temperature revealed values indicating their possible configurations as polymer–metal coordination complexes. Indeed, the magnetic moment of the manganese complex was 5.72 BM, a value exhibiting a high-spin d^5^ system engaging five unpaired electrons with a tetrahedral configuration around Mn(II) [47]. As for the magnetic moment of the iron complex, the measurement of the magnetic moment revealed a value of 5.92 BM, thus suggesting a configuration of Fe(III) ions of high-spin d^6^ with five unpaired electrons in their outer valence shell suitable for the octahedral arrangement around Fe(III) [48]. The nickel polymer complex exhibits a magnetic moment of around 2.22 BM. A corresponding value was predicted for a high-spin d^8^ system with two unpaired electrons, indicating that it has a tetrahedral geometric shape. As for the copper(II) complex, it exhibits a magnetic moment in the range of 1.84 BM, a value that is well within the expected region found for the square planar Cu(II) complex [49]. Magnetic susceptibility data coupled with the electronic spectra revealed the different complex structures shown in Figure 3.

### 3.5. Thermogravimetric Analysis

**Figure 4:**
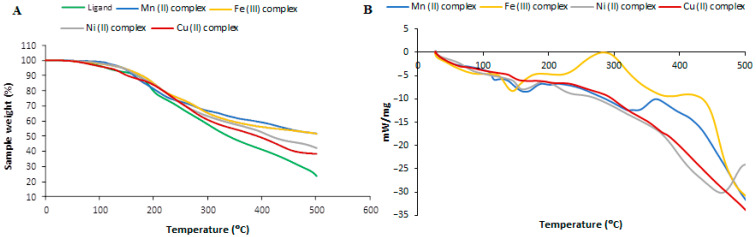
Thermograms of the ligand (poly-AG/CTR/CAR) and the different related polymeric complexes, (**A**) TGA and (**B**) DTA.

TGA-DTA thermal analysis was performed to first characterize the polymeric ligand and evaluate the thermal behavior of the synthesized cross-linked poly-AG/CTR/CAR. In a second step, this characterization will be explored to assess the thermal properties of the different polymeric complexes produced. Figure 4 and Table 4 show the TGA, DTA, and thermal decomposition results of the ligand and the different polymeric complexes. The crosslinked polymer revealed a principal step corresponding to both alginate and carrageenan biopolymers. It is important to note the relatively high weight of the residues after decomposition around 24% compared to the decomposition of alginate or carrageenan in the literature [4]. This finding is a good reflection of the success of the crosslinking reaction and the improvement in the thermal stability of the polymeric ligand after the crosslinking process. Concerning the different complexes, we noticed the same shape of the TGA curves and the presence of four and five steps of thermal decomposition. However, the different complexes showed better thermal stability compared to the ligand alone. For the manganese polymeric complex, [Mn(AG/CTR/CAR)(H_2_O)_2_], the thermal curves consisted of four main decomposition steps occurring at 66–110 °C, 112–200 °C, 202–350 °C, and 352–500 °C. The first weight-loss step started from 66 °C to 127 °C during which the weight loss is attributed to the release of the two coordinated water molecules (calculated 4.06%, found 3.10%) with a DTG midpoint at 108 °C, which is associated with an endothermic peak at 110 °C. The second stage of weight loss started at 112 until 222 °C, and was associated with a loss of the AG ligand (calculated 19.78%, found 18.63%), for which the DTG curve showed a midpoint at 163 °C and an exothermic peak at 164 °C. The third and fourth stages seemed sharp and corresponded to the decomposition of the rest of the compound with a weight loss calculated as 70.09%, and found as 70.01%. The DTG curve exhibited midpoints at 242 °C and 313 °C while the DTA showed an exothermic peak centered at 244 °C and 315 °C. The remainder of the complex decomposed in the subsequent steps, leaving a stable residue of MnO (calculated 8.01%, found 7.81%). In the case of the iron polymeric complex [Fe(AG/CTR/CAR)(H_2_O)_3_], thermal analysis revealed three distinct decompositions steps which occurred in the temperature ranges 72–112 °C, 114–206 °C, and 208–500 °C. Indeed, in the first stage, the molecules of crystalline and coordinated water are released (calculated 5.75%, found 4.55%). The corresponding DTG peak occurred at 110 °C followed by an endothermic peak which appeared at 112 °C in the DTA thermogram. The second stage indicates the expulsion of chlorine (calculated 3.77%, found 3.42%). The DTG curve displayed this step at 198 °C and the corresponding DTA exothermic peak at 199 °C. The third weight loss was assigned to the decomposition of the rest of the compound. The final remaining stable product is attributed to Fe_2_O_3_ (calculated 8.50%, found 7.28%). The thermogram of the [Ni(AG/CTR/CAR)(H_2_O)_2_] complex shows three main stages in the ranges 76–118 °C, 120–198 °C, and 200–500 °C. As with the later complexes, the first step is consistent with the release of the two coordinated water molecules (calculated 4.05%, found 4.03%) with a DTG midpoint at 97 °C which is associated with an endothermic peak at 99 °C. The observed weight loss of the second step is associated with the loss of ligand AG (calculated 19.70%, found 18.86%), for which the DTG curve presents a midpoint at 196 °C with an exothermic peak at 197 °C, which appeared in the DTA curve. The third stage corresponds to the decomposition of the rest of the complex, leaving a stable residue of NiO (8.40% accounted for, 8.22% found). The DTG curve shows a midpoint at 268 °C and 316 °C while the DTA shows an exothermic peak recorded at 270 °C and 318 °C. For the thermal behavior of the polymer complex [Cu(AG/CTR/CAR)(H_2_O)_2_], the thermogram indicates that there are three distinct stages of mass loss, namely, at 65–108 °C, 110–196 °C, and 198–500 °C. The first step is consistent with the release of the two coordinated water molecules (calculated 4.03%, found 4.01%) with a DTG midpoint at 112 °C which is associated with an endothermic peak at 114 °C. The observed mass loss from the second step is associated with the loss of ligand AG (calculated 19.59%, found 18.45%), for which the DTG curve shows a midpoint at 185 °C, and an exothermic peak at 186 °C appears in the trace DTA. The third step corresponds to the decomposition of the rest of the compound. The DTG curve shows a midpoint at 256 °C and 310 °C while the DTA exhibits exothermic peaks recorded at 258 °C and 312 °C. The remainder of the complex decomposes in subsequent steps, leaving a residue of copper oxide, CuO (calculated 8.90%, found 7.95%).

### 3.6. XRD Analysis of the Polymeric Complexes

**Figure 5:**
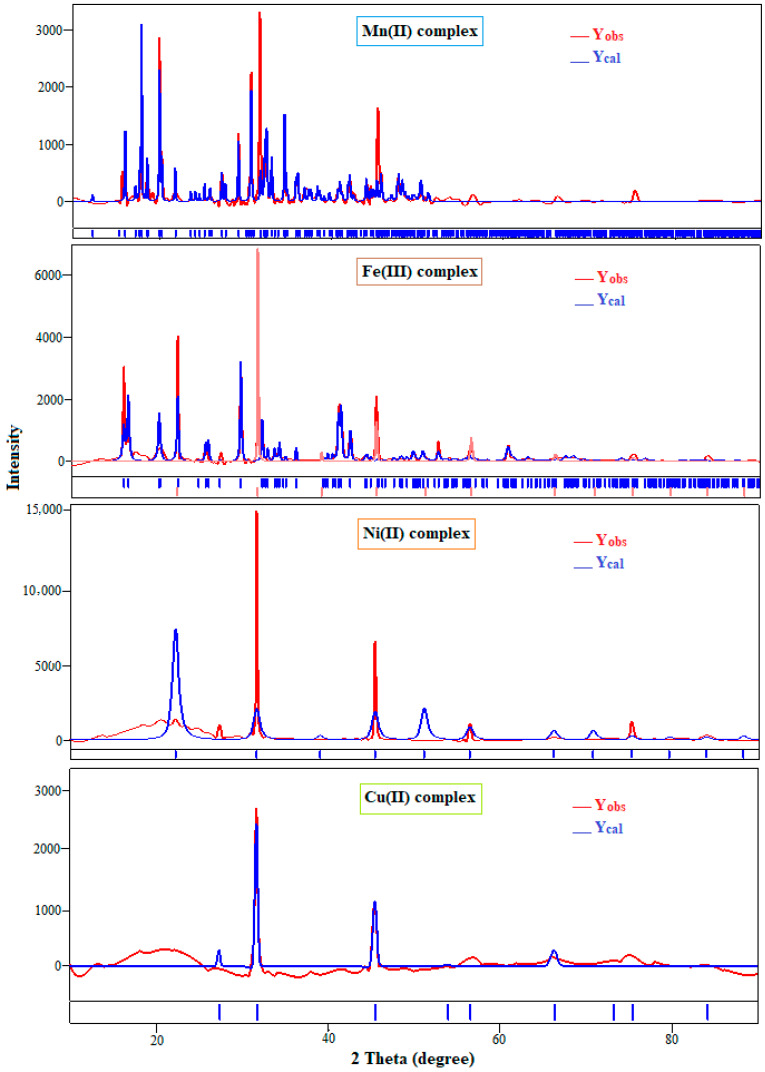
X-ray diffraction patterns of the different prepared polymeric complexes.

The different XRD patterns of the various prepared polymeric complexes are shown in Figure 5. Results revealed that the compounds are crystalline. The crystal data for the Mn(II) polymeric complex belong to the monoclinic crystal system with the space group P121/n1. For the Fe(III) and Ni(II) complexes, the crystal data fit the cubic crystal arrangement with the space group Pm-3m. The estimated data for the Cu(II) polymeric complex revealed a cubic system with the space group Fm-3m. The significant broadening of the peaks indicates that the particles are of nanometer dimensions. Scherrer’s equation was applied to evaluate the particle sizes of the polymeric complexes. An overview of the various XRD crystal data is presented in Table 5.

### 3.7. SEM Morphological Analysis

**Figure 6:**
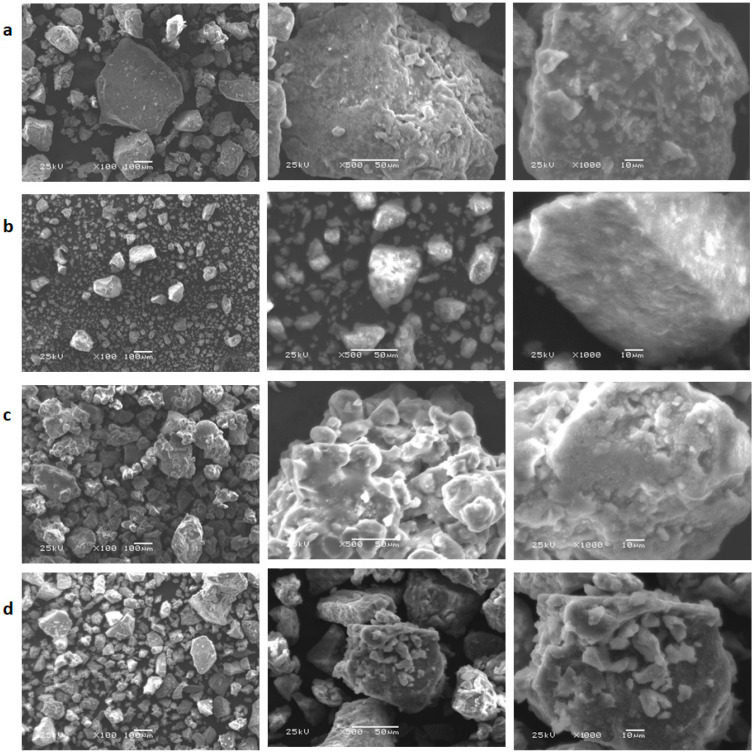
SEM micrographs of the different polymeric complexes at different magnifications: (**a**) Mn(II), (**b**) Fe(III), (**c**) Ni(II), and (**d**) Cu(II) complexes.

SEM analysis was performed to study the morphological surface characteristics of the different polymeric complexes synthesized. Different magnifications were exploited to evaluate the surface properties and the impact of each type of added metal ions. Figure 6 shows, as a general remark, a clear difference in surface morphology which varies according to the type of metal used. In all micrographs, the crosslinked polymeric ligand revealed a porous and irregular surface reflecting its significant hydrophilic property. This surface property could be effective in the formation of ligand–metal complexes and in their eventual exploitation such as biological application [50]. Figure 6a shows an irregular stalactite shape of the polymeric ligand in the presence of the Mn(II) metal. The micrometer size was randomly distributed. The surface morphology of the Fe(III) compound appears in the form of fine-sized and well-formed crystals (Figure 6b). Microscopic images of Ni(II) and Cu(II) compounds indicate the presence of irregular structures and completely different sizes from each other. Therefore, this microscopy characterization has well confirmed the different complex formations and defined the morphological behavior of each polymeric complex.

### 3.8. Biological Activity of the Different Polymeric Complexes

#### 3.8.1. Microbial Activity

**Figure 7:**
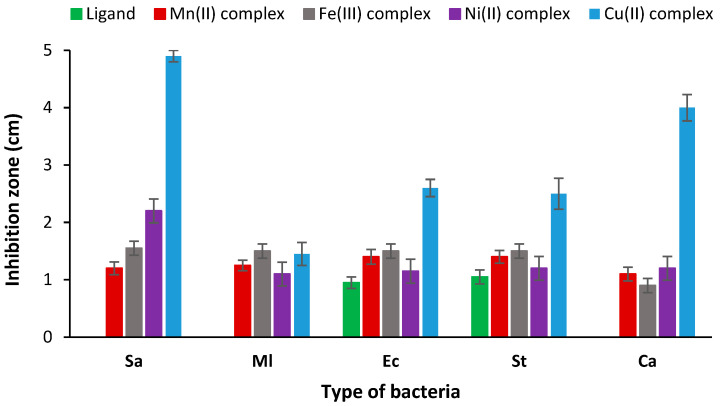
Antibacterial and antifungal evaluation of the ligand and the different polymeric complexes against various bacteria strains via the measurement of the inhibition zone around samples.

**Figure 8:**
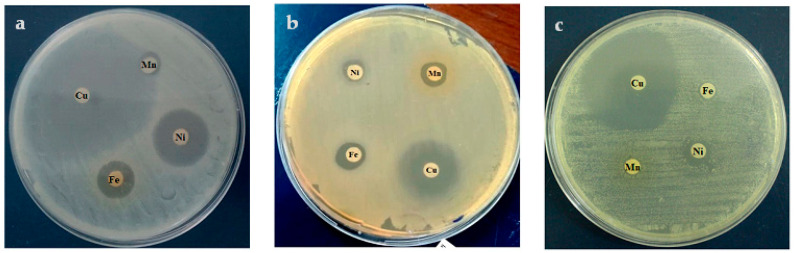
Microbiological screening of Mn(II), Fe(III) Ni(II), and Cu(II) complexes against (**a**) *S. aureus*, (**b**) *S. thyphimurium,* and (**c**) *C. Aalbicans*.

The microbiological analysis was carried out using four selected pathogenic bacteria, two of which are Gram-positive, *Staphylococcus aureus* (Sa) and *Micrococcus luteus* (Ml), and two of which are Gram-negative bacterial strains, *Escherichia coli* (Ec) and *Salmonella typhimurium* (St). The antimicrobial activity of the different complexes prepared was determined and compared to that of the polymeric ligand using the disk diffusion method. The antimicrobial activity was evaluated after direct contact with the bacterial strains, by measuring the diameter of the inhibition area that appeared around the disc samples. This zone of inhibition is directly linked to the antibacterial behavior of the sample analyzed. Results presented in Table 6 showed no activity of the ligand against the two Gram-positive bacterial strains. However, moderate activity was recorded against the two Gram-negative bacteria with a diameter of inhibition of 0.95 and 1.05 cm for Ec and St bacteria, respectively. The ligand has no fungal activity against the Ca strain. The different analyzed complexes revealed an obvious antimicrobial and antifungal activity against all the bacterial strains (Figure 7). The Cu(II) complex revealed the best microbiological results compared to the other complexes (Figure 7 and Figure 8). Indeed, excellent antimicrobial activity was recorded with an inhibition zone diameter of 4.9 and 1.45 cm against Sa and Ml bacteria, respectively, and 2.6 and 2.5 cm in the case of Ec and St strains. In addition, the antifungal effect was also excellent with 4 cm of inhibition zone. Overall, the Ni(II) polymeric complex ranked second with significant antimicrobial and antifungal effects against all bacterial strains. The antibacterial performances of the different complexes against the tested pathogenic strains were classified in the following order: Cu(II) > Ni(II) > Fe(III) > Mn(II). The excellent microbiological performances of the different polymeric complexes, using different metals, via antimicrobial and antifungal activities, confer the poly-AG/CTR/CAR/metal complexes a promising potential in the field of medical biomaterials.

#### 3.8.2. Antioxidant Activity

**Figure 9:**
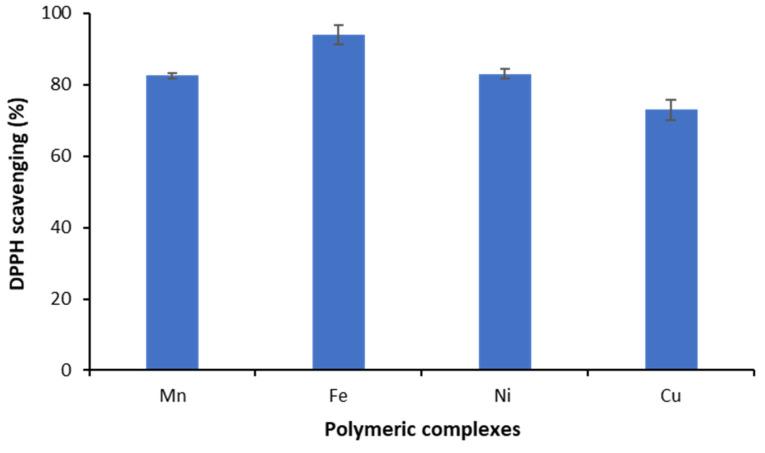
Antioxidant activity of the ligand and the different polymeric complexes.

Oxidative stress through the formation of free radicals in the body is an obvious threat to human health and it is considered a sure sign of cancer and the cause of several chronic diseases. Treatment with antioxidant compounds could scavenge these free radicals and thus reduce the risk of disease. DPPH assessment is a reliable and rapid method for easily determining antioxidant activity and is widely reported as the most effective for the antioxidant evaluation of natural and synthetic compounds [51]. As shown in Table 6 and Figure 9, the results revealed a high antioxidant potential for all the evaluated polymeric complexes. Indeed, the DPPH scavenging activity varies from 73 to 94%. The biopolymeric ligand has recorded a high antioxidant effect of 95%. 

#### 3.8.3. In Vitro Anticancer Study

**Figure 10:**
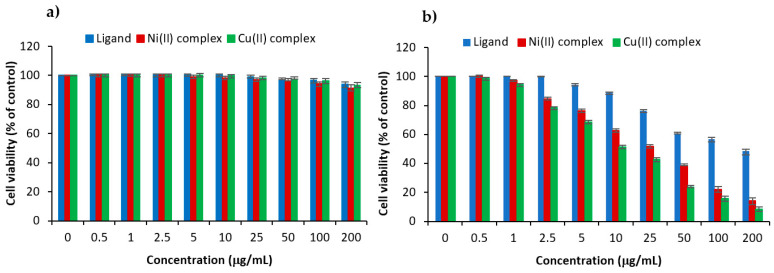
Cell viability assay in human normal beast cells (**a**) and anticancer activity in human breast cancer cells (**b**) of the ligand and the polymeric complexes via cell growth inhibition rates by varying the concentration after 72 h incubation on MCF-7 and MCF-10A cell lines.

In vitro, cell assays were performed to investigate both the biocompatibility and anticancer activity of the synthesized polymeric vectors. Human breast cancer cells (MCF-7) were used to assess the anticancer potential of the crosslinked polysaccharide ligand and its two metal complexes, the poly-AG/CTR/CAR/Cu(II) and the poly-AG/CTR/CAR/Ni(II). Different polymeric concentrations were selected in the treatment of the cancer cells and the MTT assay was performed for the evaluation of the anticancer performance. The results in Figure 10a showed no cytotoxicity of the polymeric ligand in contact with normal epithelial cells. This is in line with various reported studies that confirmed the good biocompatibility of naturally extracted polysaccharides [52,53]. Therefore, the crosslinked polymer prepared of alginate and carrageenan could be used as a safe drug delivery carrier. The two prepared polymeric complexes of Cu(II) and Ni(II), in turn, demonstrated excellent cytocompatibility with the normal human breast epithelial cells (MCF10A) with the different varied doses. This was a crucial step since the performance of anticancer drugs depends on their biocompatibility with the normal counterparts of the targeted cancer cells. The anticancer evaluation in Figure 10b showed that the polymeric ligand alone could kill cancer cells significantly from a dose of 50 μg/mL with an IC50 of 18.46 μg/mL. The anticancer performance increases with the sample concentration to reach 43% at a concentration of 200 μg/mL. This finding was consistent with other studies reported in the literature [54].

The two designed polymeric complexes revealed a significant killing potential against cancer cells in a dose-dependent manner. As in the antibacterial activity, the Cu(II) complex exhibited a higher anticancer performance with an IC50 value of 10.54 μg/mL compared to the Ni(II) polymeric complex (IC50 = 11.65 μg/mL). After 72 h of incubation and at a dose of 200 μg/mL, the metal complexation significantly decreased the viability of MCF-7 cancer cells up to 8.2 ± 1.41% and 14.2 ± 1.64% for the Cu(II) and the Ni(II) polymeric complexes, respectively. Overall, the ligand and the two complexes showed excellent cytocompatibility when in contact with normal human epithelial cells. Moreover, the in vitro anticancer study revealed the high anticancer potential of the prepared polymer complexes which crucially increased with the applied dose.

## 4. Conclusions

It is concluded that the synthesized crosslinked alginate/carrageenan biopolymer is an excellent ligand for complexing Mn(II), Fe(III), Ni(II), and Cu(II) metal cations. The various polymeric complexes formed were then characterized via IR, UV, TGA-DTA, XRD, and SEM analyses. The microbiological assessment revealed excellent antibacterial and antifungal potentials of the various polymeric complexes. The Cu(II) complex showed higher activity against both the two Gram-positive and Gram-negative bacteria strains. The current study demonstrated that all of the polymer complexes were good sources of antioxidants. Indeed, the DDPH analysis showed the ability of the four complexes to significantly scavenge free radicals with a DPPH scavenging activity varying from 73 to 94%. Viability assays demonstrated the good cytocompatibility of the polymeric crosslinked ligand of alginate and carrageenan which could be applied as an effective and safe drug delivery carrier. Cu(II) and Ni(II) polymer complexes exhibited no cytotoxicity in contact with normal human breast epithelial cells (MCF10A). Moreover, they showed anticancer activity with human breast cancer cells (MCF-7) which increases significantly with dose, with an IC50 of 10.54 and 11.65 μg/mL, respectively. Additional biological tests to be carried out in the future will still be able to support our results and further prove the interest in our polymeric complexes. Further research on the antitumor performance of the poly-AG/CTR/CAR/Cu(II) and poly-AG/CTR/CAR/Ni(II) complexes in animal models is warranted.

## Figures and Tables

**Table 1:** Color, elemental analysis, and decomposition point of the complex biopolymers.

Complex	M.F (M.Wt)	Color	Found (Calculated %)			m.p.C° Decomp.	Λm Scm^2^ mol^−1^
			C	H	S		
Ligand (poly-AG/CTR/CAR)	C_24_H_27_O_26_S_2_	Dark	36.92	3.76	8.52	110	-
	(795.37)	Beige	(36.23)	(3.42)	(8.06)		
[Mn(AG/CTR/CAR)(H_2_O)_2_]	C_24_H_29_O_28_S_2_Mn	Light-Brown	33.05	3.62	7.98	150	2.58
	(884.54)		(32.59)	(3.30)	(7.25)		
[Fe(AG/CTR/CAR)(H_2_O)_3_Cl]	C_24_H_31_O_29_S_2_FeCl	Light-Orange	29.86	3.11	6.93	155	2.08
	(938.90)		(30.69)	(3.32)	(6.82)		
[Ni(AG/CTR/CAR)(H_2_O)_2_]	C_24_H_29_O_28_S_2_Ni	Light- Green	34.07	4.06	7.86	162	6.78
	(888.29)		(32.45)	(3.29)	(7.22)		
[Cu(AG/CTR/CAR)(H_2_O)_2_]	C_24_H_29_O_28_S_2_Cu	Light-Blue	33.12	3.90	7.75	166	2.95
	(892.95)		(32.27)	(3.27)	(7.18)		

**Table 2:** FTIR spectral bands and their assignments of the ligand and its metal complexes.

Compound	ᵛ(O-H)	ᵛ(C-H)	ᵛ(C=O)	ᵛ(C=C)	ᵛ(S=O)	ᵛ(C-O)	ᵛ(=C-H)	ᵛ(-C-H)	ᵛ(M-O)
Ligand (poly-AG/CTR/CAR)	3431	2177	1725	-	1260	1035	783	560	-
[Mn(AG/CTR/CAR)(H_2_O)_2_]	3315	-	1699	1598	1206	1026	928	700	508
[Fe(AG/CTR/CAR)(H_2_O)_3_Cl]	3380	-	1716	1610	1214	1027	926	840	516
[Ni(AG/CTR/CAR)(H_2_O)_2_]	3260	2917	1715	1614	1395	1025	926	787	510
[Cu(AG/CTR/CAR)(H_2_O)_2_]	3353	2917	1715	1583	1214	1025	927	864	505

**Table 3:** UV–Vis spectra and magnetic moments values of the ligand and its metal complexes.

Complex	Vmax (nm)	Assignment	µeff. (BM)
Ligand (poly-AG/CTR/CAR)	24.213	n→π*	-
	39.215	π→π*	
[Mn(AG/CTR/CAR)(H_2_O)_2_]	29.761	n→π*	5.72
	35.842	π→π*	
	21.551	d–d transition	
[Fe(AG/CTR/CAR)(H_2_O)_3_Cl]	29.411	n→π*	5.92
	32.573	π→π*	
	21.598	d–d transition	
[Ni(AG/CTR/CAR)(H_2_O)_2_]	23.202	n→π*	2.20
	34.072	π→π*	
	21.978	d–d transition	
[Cu(AG/CTR/CAR)(H_2_O)_2_]	24,331	n→π*	1.84
	36,221	π→π*	
	21,739	d–d transition	

**Table 4:** Thermal decomposition data for the different polymeric complexes.

Complex	Temp. Range (°C)			TGA (Wt. Loss%)		Assignment
	T_i_	T_m_	T_f_	Calculated	Found	
Mn(II) complex	66	110	127	4.06	3.10	Loss of two water molecules.
	112	200	222	19.78	18.63	Loss of ligand (AG).
	202	350	313	70.09	70.01	Decomposition of the rest of the organic ligands with the formation of MnO.
	352	500	550	8.01	7.81	
Fe(III) complex	72	112	146	5.75	4.55	Loss of three water molecules.
	114	206	240	3.77	3.42	Loss of chloride atom.
	208	500	550	8.50	7.28	Decomposition of the rest of the organic ligands with the formation of (½ Fe_2_O_3_).
Ni(II) complex	76	118	150	4.05	4.03	Loss of two water molecules.
	120	198	248	19.70	18.86	Loss of ligand (AG).
	200	500	550	8.40	8.22	Decomposition of the rest of the organic ligands with the formation of NiO.
Cu(II) complex	65	108	128	4.03	4.01	Loss of two water molecules.
	110	196	181	19.59	18.45	Loss of ligand (AG).
	198	500	550	8.90	7.95	Decomposition of the rest of the organic ligands with the formation of CuO.

T_i_ = Initial temperature, T_m_ =Maximum temperature, T_f_ = Final temperature.

**Table 5:** XRD Crystal data of the different polymeric complexes (R_wp_(%) = 6.49, R_exp_(%) = 4.31, and R_B_(%) = 1.03).

Parameters	Mn(II) Complex	Fe(III) Complex	Ni(II) Complex	Cu(II) Complex
Empirical formula	C_24_H_29_O_28_S_2_Mn	C_24_H_31_O_29_S_2_FeCl	C_24_H_29_O_28_S_2_Ni	C_24_H_29_O_28_S_2_Cu
Formula weight	884.54	938.90	888.29	892.95
Crystal system	Monoclinic	Cubic	Cubic	Cubic
a (Å)	11.197	3.9908	3.9940	5.628
b (Å)	9.520	3.9908	3.9940	5.628
c (Å)	6.195	3.9908	3.9940	5.628
Alfa (°)	90.00	90.00	90.00	90.00
Beta (°)	99.74	90.00	90.00	90.00
Gamma (°)	90.00	90.00	90.00	90.00
Volume of unit cell (Å^3^)	650.9	63.56	63.71	178.29

a, b, and c are the dimensions of the crystal system. Alfa, Beta, and Gamma are the angles of the crystal system of the different complexes.

**Table 6:** Antimicrobial and antioxidant activity of the ligand and the different polymeric complexes.

	Antimicrobial Activity (Inhibition Zone (cm))					Antioxidant
	Sa	Ml	Ec	St	Ca	
Ligand	0	0	0.95	1.05	0	95^±1.4^
Mn(II) complex	1.2	1.25	1.4	1.4	1.1	82.5^±0.7^
Fe(III) complex	1.55	1.5	1.5	1.5	0.9	94^±2.8^
Ni(II) complex	2.2	1.1	1.15	1.2	1.2	83^±1.4^
Cu(II) complex	4.9	1.45	2.6	2.5	4	73^±2.8^

## Data Availability

The data are contained within the article.
